# Birth Preparedness and Complication Readiness among Slum Women in Indore City, India

**DOI:** 10.3329/jhpn.v28i4.6045

**Published:** 2010-08

**Authors:** Siddharth Agarwal, Vani Sethi, Karishma Srivastava, Prabhat K. Jha, Abdullah H. Baqui

**Affiliations:** ^1^ Urban Health Resource Center, New Delhi, India; ^2^ Formerly with Urban Health Resource Center, New Delhi, India; ^3^ Urban Health Resource Center, Indore, India; ^4^ International Center for Advancing Neonatal Health, Department of International Health, Johns Hopkins Bloomberg School of Public Health, Johns Hopkins University, Baltimore, MD 21205, USA

**Keywords:** Birth preparedness, Complication readiness, Cross-sectional studies, Deliveries, Slums, Urban poor, India

## Abstract

Three hundred twelve mothers of infants aged 2-4 months in 11 slums of Indore, India, were interviewed to assess birth preparedness and complication readiness (BPACR) among them. The mothers were asked whether they followed the desired four steps while pregnant: identified a trained birth attendant, identified a health facility, arranged for transport, and saved money for emergency. Taking at least three steps was considered being well-prepared. Taking two or less steps was considered being less-prepared. One hundred forty-nine mothers (47.8%) were well-prepared. Factors associated with well-preparedness were assessed using adjusted multivariate models. Factors associated with well-preparedness were maternal literacy [odds ratio (OR)=1.9, (95%) confidence interval (CI) 1.1-3.4] and availing of antenatal services (OR=1.7, CI 1.05-2.8). Deliveries in the slum-home were high (56.4%). Among these, skilled attendance was low (7.4%); 77.3% of them were assisted by traditional birth attendants. Skilled attendance during delivery was three times higher in well-prepared mothers compared to less-prepared mothers (OR: 3.0, CI 1.6-5.4) Antenatal outreach sessions can be used for promoting BPACR. It will be important to increase the competency of slum-based traditional birth attendants, along with promoting institutional deliveries.

## INTRODUCTION

High levels of perinatal (49 per 1,000 births), neonatal (39 per 1,000 livebirths) (1), and maternal mortality (301 per 100,000 livebirths) (2) remain major public-health challenges in India. About one-third of neonatal deaths occur on the first day of life (1), and the majority of maternal deaths occur during labour, delivery, and within 24 hours postpartum (3). Apart from medical causes, there are numerous interrelated sociocultural factors which delay care-seeking and contribute to these deaths. Care-seeking is delayed because of the delay in (a) identifying the complication, (b) deciding to seek care, (c) identifying and reaching a health facility, and (d) receiving adequate and appropriate treatment at the health facility (4). Birth preparedness and complication readiness (BPACR) is one intervention that addresses these delays by encouraging pregnant women, their families, and communities to effectively plan for births and deal with emergencies, if they occur. At the basic level, the concept of BPACR includes identifying a trained birth attendant for delivery, identifying a health facility for emergency, arranging for transport for delivery and/or obstetric emergency, and saving money for delivery (5). There is evidence from rural Nepal (6), Burkina Faso (7), Ethiopia (8), and India (8,9) that promoting BPACR improves preventive behaviours, improves knowledge of mothers about danger-signs, and leads to improvement in care-seeking during obstetric emergency. However, no data are available on levels of BPACR among the urban poor in India.

An estimated 340 million of 1.1 billion people in India live in cities. About 100 million city-dwellers live in slums, pavements, construction sites, or urban fringes in extreme poverty (10). As many of the urban poor live in temporary settlements and slums not included in the government official slum lists, they are often excluded from basic government services and, as a result, constantly struggle for housing, livelihood, and healthcare. This compromises their capacity to adequately care for pregnant and delivering women. Several socioeconomic factors also limit their ability to seek requisite healthcare, despite their geographical proximity to health facilities (11). This paper presents the levels and factors associated with BPACR among slum women of a city in India, including the use of skilled care provider at birth, and provides recommendations for urban programmes.

## MATERIALS AND METHODS

### Study design and site

This cross-sectional study was conducted in 11 slums in Indore city in the state of Madhya Pradesh, India. Madhya Pradesh is situated in the central part of India—far from the sea. Area-wise, it is the second largest Indian state and is the home to 60 million people. Indore is one of the 48 districts of Madhya Pradesh and the home to over two million people (12). Over two-thirds (70%) of its population reside in urban areas or cities (12). Being the financial capital of the State, Indore city attracts migrants from adjoining hinterlands and other states of India. Uncontrolled and unplanned growth of this urban centre and a large and fast-growing slum-dwelling population city has led to the city's infrastructure being under significant stress. Over 40% of residents in this city live in poverty, in slums and squatters, characterized by substandard and squalid housing and lacking inadequate access to basic civic facilities. A situation analysis of the city in 2003 showed that there were 539 slums, of which 105 were not even recognized by the Government, which made them remain outside the purview of basic government health and civic services (11).

The health service-delivery system in Indore city comprises a multi-tiered system of dispensaries, second-tier hospitals, and tertiary referral hospitals. At the time of initiation of this study, public-sector health services in Indore city comprised one tertiary-care hospital (associated with the district government medical college), one district hospital, three small maternity hospitals, and 17 functional dispensaries/urban family welfare centres. In only two of the above facilities, round-the-clock emergency obstetric care services were provided.

Primary health services, especially to the urban poor of Indore city, are grossly inadequate. The size of the target population covered by a health worker (auxillary nurse midwife or ANM) responsible for providing outreach services approximates 20,000-25,000, which is far greater than government norms. This contributes to either slums not being covered by health services or receiving irregular and suboptimal outreach services. The private informal sector is, by far, the most preferred source of treatment and consists predominantly of unqualified medical practitioners (11).

The 11 slums where the present study was conducted were among the 79 slums where a maternal-child health programme of a non-governmental organization (NGO)—Urban Health Resource Centre (UHRC)—has been operational since 2003. The programme has been described in detail elsewhere (13). With technical support from the UHRC, five Indore-based NGOs implement the programme through a network of slum-based female groups, health volunteers (1 per 3,000 people), and field supervisors (1 per 15,000 people). As part of the programme, slum-based female groups facilitated by NGOs were also encouraged to save collectively each month to form a community health fund. The promotion of BPACR is one of the several maternal-child health behaviour-change interventions which was introduced 2-3 months before the initiation of the study in response to programme needs. Health volunteers had undergone one round of training by the programme staff to promote BPACR during monthly antenatal service-coverage camps and monthly meetings of mothers’ group.

The 11 slums were purposively selected for the present study after consulting the field supervisors. These slums were comparable with regard to the approximate proportion of deliveries in the home (at least half), poverty (half of all families dependent on daily-wage), most to moderate category health vulnerability (14), being at a distance of 7-10 km from a government maternity-care facility, with a private health facility in closer proximity, counselling by health volunteers, and organization of monthly antenatal outreach camps with coordination with government functionaries in all the 11 slums. The total population in these 11 slums at the time of initiation of this study was 24,395.

Two trained social science postgraduates not involved in any programme activities collected data during December 2004–February 2006. Data-collectors were imparted a two-day training, which involved class-room sessions and hands-on learning in the field. Collection of data was initiated only after both the data-collectors demonstrated adequate competence in eliciting consistent responses based on 10 interviews each. The second author of the study supervised the collection of data.

A mother whose youngest child was a live infant and aged 2-4 months was included as the sample. There were no twin births in the sample. The sample-size was calculated to estimate a hypothesized prevalence of 50% with ±10% precision using a standard one sample formula. The sample-size was inflated assuming a design effect of 1.5 and a non-response rate of 10%. The final sample-size was 321. We hypothesized 50% prevalence for BPACR indicators as no prior estimates were available, and an assumed 50% prevalence provided the largest sample-size.

Samples were enlisted using birth-record registers of the health volunteers. These registers were maintained by the health volunteers who were supervised by NGO staff who ensured that registers were adequately filled. The health volunteers tracked pregnancies and births during regular meetings of mothers in each slum. Barring about 10% of births where the woman went to her native village for delivery, approximately 90% of all births were recorded in the registers. Visits to home were made for data collection from the enlisted mothers until the required sample-size was covered. In total, 321 mothers were visited, and complete data were collected from 312 of them.

Using a pretested interview schedule, the following aspects were enquired from the mothers in Hindi: (a) family characteristics, including religion, caste, family-size, educational level of mothers, and occupation and educational level of husbands; (b) pregnancy characteristics, including parity; (c) whether the mother availed of the following antenatal services while pregnant: received two tetanus toxoid (TT) injections, received at least three antenatal check-ups (ANCs), received iron folic acid (IFA) tablets, consumed IFA tablets for at least three months; antenatal services were received either during monthly antenatal outreach sessions/camps conducted by trained paramedic (ANM or medical professional) or during ANC visit to health facility; (d) awareness of pregnancy, delivery and newborn danger-signs indicative of referral, and (e) whether the mother followed the following four most basic BPACR practices (5) while pregnant: (i) identified a trained birth attendant for delivery, (ii) identified a health facility for emergency, (iii) arranged for transport for delivery and/or obstetric emergency, and (iv) saved money.

Reasons for not following each of the four BPACR practices were also enquired during the interview.

All the mothers were asked about the place of their delivery (slum-home, native-village, or health facility) and birth attendant who conducted the delivery.

If the husband was unemployed or a daily-wage labour or was a rickshaw-puller, the occupation was coded as an irregular job. If the husband was a driver or a small business-owner or employed in a private job, the occupation was coded as a regular job. If the delivery was conducted in a health facility or in the home by a nurse, an ANM, or a doctor, the birth attendant was categorized as skilled. If a trained traditional birth attendant (TBA) conducted delivery in the home, delivery was considered being conducted by a trained birth attendant. Deliveries conducted by neighbours, family members, or untrained TBAs were considered being conducted by untrained birth attendants. There were 37 slum-based TBAs (henceforth called sTBAs) in the 11 slums. All the 37 sTBAs were illiterate, middle-age women providing delivery services for five years or more and had conducted at least one delivery in one year preceding the enquiry. They belonged to the schedule or backward class. Of these, 29 had attended one or more training programmes on optimal delivery practices hosted by NGOs/doctors in government hospitals ever since they began their work. These 29 TBAs were considered trained TBAs. Background information about TBAs was enquired from all the 37 TBAs themselves by the data-collectors.

Unprompted spontaneous responses were sought from mothers on whether they faced any complication during delivery. The self-reported conditions were enquired in local terminology. These were then related to medical terminologies (given in parenthesis) and included: excessive vaginal bleeding, high fever, vaginal discharge, convulsions, prolonged labour (labour that lasted for over 12 hours), obstructed labour (malpresentation), and retained placenta (all or part of the placenta or membranes are left behind in the uterus beyond 30 minutes). Those mothers who reported a complication were asked which birth attendant they approached during this time.

### Statistical analysis

Collected data were consolidated on Excel sheets. The consolidated data were re-checked for completeness and accuracy.

First, standard descriptive analysis was carried out. Then, those mothers who followed at least three of the four BPACR practices were considered ‘well-prepared’. The remaining mothers were considered ‘less-prepared’. This approach of categorizing well-prepared and less-prepared mothers has also been used earlier in a study on birth preparedness (9).

The association of family characteristics, pregnancy characteristics, and maternal knowledge with BPACR was examined using chi-square test for homogeneity. The net association of these variables was then examined in a multivariate analysis using binary logistic regression adjusting for covariates considered to influence the outcome.

Finally, whether being well-prepared vs less-prepared had an association with skilled attendance during delivery was assessed using chi-square test and multivariate analysis using binary logistic regression.

Analysis of data was conducted using the Stata software (version 9.1) (Stata Corporation, College Station, TX, USA). The p value of <0.05 was considered statistically significant.

## RESULTS

### Sample characteristics

The study sample comprised predominantly Hindus (97.8%) and backward castes (90.7%). Illiteracy rates were high (67.9% of mothers and 40.4% of fathers). The mean family-size was six. Over three-fourths (75.6%) of the fathers did not have regular jobs. Since the study was conducted after one year of programme activities in these slums, receipt of antenatal services, such as 2 TT injections, 3 ANCs, and IFA tablets was not low, being 82%, 40.1%, and 86.2% respectively. However, only 28.5% of the mothers consumed IFA tablets for 3+ months during pregnancy. Awareness of the mothers about at least one danger-sign of pregnancy, delivery, and newborn-related complications was not also low, being 79.2%, 78.5%, and 82.1% respectively. Nearly three-fourths of the deliveries (n=225) took place in the home (56.4% in slum-homes and 15.7% in native-village). Of these deliveries in the slum-home, skilled birth attendants assisted in only 7.4% of the deliveries; 77.3% of the deliveries were attended by TBAs. Overall, only 32% of the deliveries (home and health facility combined) were attended by skilled birth attendants (Table 1).

**Table 1.** Selected characteristics of study sample

Characteristics	No.	%
Sociodemographic characteristics		
Religion		
Muslim	7	2.2
Hindu	305	97.8
Caste		
Upper—General	29	9.3
Lower—Schedule caste/tribe	132	42.3
Other backward caste	145	46.5
Maternal illiteracy	212	67.9
Paternal illiteracy	126	40.4
Family income		
Irregular*	236	75.6
Regular†	76	24.4
Family-size (mean, SD: 6,2)		
≤4	87	27.9
5-6	118	37.8
7 or more	107	34.3
Pregnancy characteristics		
Parity		
1	85	27.2
2-3	163	52.2
4-9	64	20.5
Took 2 TT shots	256	82.0
Took 3 or more ANC check-ups	125	40.1
Received IFA tablets	269	86.2
Consumed IFA tablets for 3 or more months	79	28.5
Availed of antenatal services (2 TT shots and 3 ANCs and received IFA tablets)	110	35.2
Aware of at least one danger-sign		
During pregnancy	247	79.2
During delivery	78.5	245
Of newborn	255	81.7
Place of birth		
Home-slum	176	56.4
Home-maternal village	49	15.7
Health facility (government/private)	87	27.9
Person who assisted births in slum-home		
Skilled attendant (paramedical nurse/doctor)	13	7.4
Traditional birth attendant	104	59.1
Untrained traditional birth attendant	32	18.2
Family member/neighbour/self	27	15.3

*Labour (skilled/unskilled) and rickshaw-puller;

†Driving, private job, and small business (sells vegetables/fruits);

ANC=Antenatal care;

ANCs=Antenatal care check-ups;

IFA=Iron folic acid;

SD=Standard deviation;

TT=Tetanus toxoid

Of the 312 mothers, 52 (16.7%) experienced delivery-related complication(s) cited earlier; 33 (63.5%) of the 52 mothers were delivered by a skilled birth attendant.

### Levels of birth preparedness and complication readiness

Over two-thirds (69.6%) of the mothers identified a trained birth attendant for delivery (Table 2). Of those mothers who did not identify a trained birth attendant for delivery (30.4%), the most predominant reasons reported were: lack of perceived need (19.8%), economic constraints (4.5%), and faith in TBAs or traditional system of delivery (6.1%).

**Table 2.** Birth preparedness and complication readiness among slum women (n=312) in Indore, India

Levels of birth preparedeness and complication readiness	No.	%
No. of mothers who report to have done the following, while pregnant		
Identified a trained birth attendant for delivery	217	69.6
Identified a health facility for emergency	199	63.8
Arranged for transport	92	29.5
Saved money	240	76.9
No. of steps taken		
0	12	3.8
1	55	17.3
2	97	31.1
3	97	31.1
4	52	16.7
At least 3 steps taken	149	47.8

About two-thirds (63.8%) of the mothers identified a health facility for obstetric emergency. The health facilities identified included private charitable hospitals (39.7%) and private nursing homes/practitioners (24.1%). Thus, the government health facilities were not the preferred choice for obstetric emergency. The mothers (36.2%) who did not identify a health facility mentioned that they did not face any complication while pregnant and, hence, planned that their delivery be conducted in the home.

The large majority (76.9%) of the families saved some money and kept it aside for incurring cost of delivery and obstetric emergencies, if needed. For the remaining 23.1% of the families, meagre earnings which were mostly spent on household purchases (16.7%) or by husband on liquor (6.4%) were cited as reasons for not saving money. Of the 312 mothers, 8% mentioned being members of the community health fund groups. The reasons cited by the remaining 92% of the mothers for not joining a community health fund group were: economic constraints (24.8%), lack of awareness about such a group (51.2%), and having no established faith in the group (16%).

Preparedness for transport for emergency was low (29.5%). It emerged from the responses of the mothers that prior arrangement for transport was not considered crucial due to the easy availability of local transport in their slum and vicinity.

Overall, 47.8% of the mothers were well-prepared, and 52.2% were less-prepared.

Compared to the less-prepared mothers, the well-prepared mothers tended to be literate, had a literate husband, availed of antenatal services, and had better knowledge about maternal/newborn danger-signs suggestive for seeking referral. The adjusted multivariate model showed that significant predictors for being well-prepared were maternal literacy (OR=1.9, CI 1.1-3.4) and availing of antenatal services (OR=1.7 CI 1.05-2.8) (Table 3). Skilled attendance during delivery was three times higher in well-prepared mothers compared to less-prepared ones (OR: 3.0, CI 1.6-5.4) even after adjusting for all the family and pregnancy characteristics. When the analysis was restricted to those mothers who reported a delivery-related complication, a higher proportion of well-prepared mothers vs less-prepared mothers had a skilled birth attendant assisting their delivery (73.9% vs 51.7%); however, this difference was not significant (Table 4).

**Table 3.** Selected characteristics of mothers who were well-prepared* vs those who were less-prepared (n=312)

Independent variable	%		Chi-square p value	Unadjusted OR (95% CI)	Adjusted OR** (95% CI)
	Less-prepared (n=163)	Well-prepared (n=149)			
Family characteristics					
Maternal literacy					
No	77.3	57.7		1.00	1.00
Yes	22.7	42.3	0.000	2.5 (1.5-4.1)†	1.9 (1.1-3.4)†
Paternal literacy					
No	46.0	34.2		1.00	1.00
Yes	54.0	65.8	0.02	1.6 (1.03-2.6)	1.41 (0.8-2.3)
Occupation of fathers					
Irregular job	77.9	73.2		1.00	1.00
Regular job	22.1	26.8	0.19	1.3 (0.7-2.2)	1.2 (0.7-2.1)
Family-size					
7 or more	33.7	34.9		1.00	1.00
5-6	38.7	36.9		0.9 (0.5-1.5)	1.06 (0.6-1.8)
≤4	50.3	28.2	0.95	1.0 (0.6-1.7)	1.00 (0.5-1.9)
Pregnancy characteristics					
Parity					
4-9	24.5	16.1		1.00	1.00
1	25.2	29.5		1.7 (0.9-3.5)	1.2 (0.6-2.5)
2-3	50.3	54.4	0.17	1.6 (0.9-2.9)	1.2 (0.6-2.4)
Availed antenatal services***					
Yes	71.2	57.7		1.00	1.00
No	28.8	42.3	0.009	1.8 (1.1-2.9)‡	1.7 (1.05-2.8)‡
Aware of at least one danger-sign during pregnancy¶					
No	25.2	16.1		1.00	1.00
Yes	74.8	83.9	0.03	1.7 (1.09-3.1)	1.5 (0.8-2.8)
Aware of at least one danger-sign during delivery§					
No	25.8	16.8		1.00	1.00
Yes	74.2	83.2	0.03	1.7 (0.98-2.9)	1.5 (0.8-2.8)
Aware of at least one newborn danger-sign††					
No	22.1	13.4		1.00	1.00
Yes	77.9	86.6	0.04	1.8 (1.1-3.2)‡	1.4 (0.7-2.6)

*Any 3 of 4 steps: identified trained birth attendant for delivery, identified health facility for emergency, arranged for transport and saved money;

**Adjusted for all the independent variables;

***Received 2 TT injections, 3 ANCs, and IFA tablets;

†p<0.01;

‡p<0.05;

¶Bleeding from the vagina before 37 weeks, severe pain in the abdomen, breathlessness, swelling on face/body, reduced foetal movements, high fever, malpresentation;

§Slow progress of labour >12 hours, excessive bleeding from the vagina, high fever accompanied by blurring of vision, fits severe pain in abdomen, early rupture of bag of water;

††Newborn is small in size/thin/skinny, lethargic, does not accept feeds, cold to touch, difficulty in breathing (grunt or severe chest in-drawing), umbilical sepsis, and very high fever;

ANCs=Antenatal care check-ups;

CI=Confidence interval;

IFA=Iron folic acid;

OR=Odds ratio;

TT=Tetanus toxoid

**Table 4.** Use of skilled birth attendance by mothers who were well-prepared* vs those who were less-prepared (n=312)

Skilled attendance during delivery (well-prepared mothers vs less-prepared mothers)	%		Chi-square p value	Adjusted OR† (95% CI)
	Less-prepared (n=163)	Well-prepared (n=149)		
Delivery attendant, all deliveries				
Skilled**	23.9	42.9		
Trained	39.3	42.3		
Untrained	36.8	14.8	0.000	3.0 (1.6-5.4)††
Delivery attendant, deliveries with delivery-related complication(s)***	(n=29)	(n=23)		
Skilled**	51.7	73.9		
Trained	27.6	17.4		
Untrained	20.7	8.7	0.24	-

*Practised any 3 of 4 steps: identified trained birth attendant for delivery, identified health facility for emergency, arranged for transport, and saved money;

**Delivery was conducted in a health facility or by a nurse, doctor, or auxillary nurse/midwife in the home;

***Reported to have had at least one of the following during delivery: excessive vaginal bleeding, high fever, bad smelling vaginal discharge, convulsions, prolonged labour (>12 hours), obstructed labour/malpresentation, and/or retained placenta;

†Adjusting for maternal illiteracy, paternal illiteracy, occupation of fathers, family-size, parity, availed of antenatal services, awareness of pregnancy, delivery and newborn danger-signs;

††p<0.0001;

CI=Confidence interval

## DISCUSSION

Several important findings emerged from this study. Less than half of the slum mothers were well-prepared for delivery and obstetric emergency; maternal literacy and availing of antenatal services were important predictors of birth preparedness; and birth preparedness was positively associated with skilled birth attendance. These findings reinforce the widely-held notion that BPACR should be promoted during pregnancy in settings where deliveries in the home are common (5).

A woman who is educated is able to make informed decisions about her own health compared to her illiterate counterpart (15). Slum-level health workers or volunteers should include literate women in meetings of female group to build confidence of illiterate women through sharing of experiences. Pictorial cards, video-films, and case narratives can be used during the meetings of female group to ease the understanding of BPACR messages being promoted (6,8).

The finding that availing of antenatal services (either through monthly antenatal service-coverage camps or through a health facility) was associated with better BPACR has immense programmatic relevance. Women who received ANCs possibly also received some form of counselling on BPACR and, hence, were more likely to prepare for birth and emergencies. Thus, although an ANC may not always lead to identifying women who are most in need of obstetric care, it can be an effective mechanism to promote better BPACR and, in turn, improve the use of skilled care at birth. This is one potential model for promoting BPACR. Thus, during antenatal outreach sessions, women should be counselled on BPACR by community-level government service providers, who can, in turn, be trained and supervised by NGOs/primary-level government service providers to play this pertinent role. Geographically-proximate health facilities providing quality services; other skilled birth attendants who can be approached for assisting delivery in the home; availability of a community health fund can also be discussed during these interactions.

Arranging for transport did not emerge as an important issue in the study slums. However, depending on local situations arranging for transport could be an important issue for slums in certain locations or cities. Hence, programmes need to tailor their BPACR messages for the context they are operating.

Delivery in the home was a common feature in the study community. Similar trends have been reported from other slum-based studies in India (16,17). Furthermore, while about 80% of the study mothers were aware of maternal/newborn-related complications, and about 50% of them reported being well-prepared, yet only 32% of the mothers used services of a skilled birth attendant. Thus, while improving knowledge and helping mothers to prepare for birth and emergencies is important, perseverant efforts are required to address the barriers that hinder skilled birth attendance and use of health facility for delivery. Since a large proportion of slum women still rely on slum-based TBAs, the latter need to be trained and monitored to effectively detect and manage complications. Slum-based TBAs can also be linked to proximate public or private accredited health facilities providing low-cost quality services so that they can recommend or even escort mothers to these facilities at times of need (18). A Government of India scheme—*Janani Suraksha Yojana*—introduced in Madhya Pradesh in late 2006 integrates cash incentive for delivery at the government or accredited private health facilities for pregnant women below poverty-line and for the health worker who escorts the pregnant woman to these facilities (19). The sTBAs can be linked with the health facilities under this scheme to enhance access to institutional delivery for slum women and help them and sTBAs avail of its benefit.

The finding of low preference for the government health facilities during obstetric emergencies in the present study highlights the need for making efforts to improve the quality of care in the government facilities. Another viable option suggested in the draft document of the Government of India's soon-to-be-launched National Urban Health Mission is identifying and tapping potential private partners and contracting delivery of selected health services to those private partners ensuring a pro-poor approach (20). Such partnerships should aim at using skills of partners, encouraging pooling of resources, and complementing investment of the Government in health ensuring that the pro-poor focus is retained.

Access to loans through the community health funds of slum-based groups may be an option. However, results of a study in Nigera showed that, while the availability of loans for emergency obstetric care through community health funds increased service-use, repayments were not always completed (21). Hence, it is crucial to identify and stimulate slum individuals with a social responsibility inclination to form community groups. Community groups once formed should be encouraged to discuss and determine the need for such a community health fund before encouraging them to begin generating and managing collective savings. It is important to stimulate them to evolve their rules to administer the health fund rather than dictate a uniform system to facilitate ownership of decisions (22). The National Urban Health Mission also envisages introducing a community health fund scheme with seed money in the first year and subsequent annual incentives to slum-based groups (20).

### Limitations

This study was one of the first slum-based studies on BPACR. However, it had several limitations, and hence, its findings should be interpreted in light of these limitations. First, the sample-size was small. Second, although the 11 study slums were comparable, they were purposively selected from all the slums in Indore. Third, this study was conducted after the start of the programme (though BPACR as an intervention was introduced only 2-3 months before the study), there could have been some collateral benefit and, hence, may reflect a situation better than other slums. Fourth, the findings are self-reported; therefore, there can be some recognition and recall error. To minimize recall errors, we selected mothers of infants aged 2-4 months. Finally, this study included mothers of surviving infants (aged 2-4 months) only; practices of mothers whose baby died could have been different.

### Conclusions

We observed that BPACR was positively associated with improved skilled birth attendance. However, despite generally high BPACR, 56.4% of the deliveries in the slum-home and 77.3% of the deliveries in the slum-home attended by slum-based TBAs highlight the need to increase the competency of slum-based TBAs to effectively detect and manage delivery-related complications while making complementary efforts to increase demand for facility-based deliveries through government schemes providing incentives to promote institutional deliveries and improving the quality of services provided in such facilities. Antenatal outreach camps are good opportunities to discuss and address barriers that hinder adoption of appropriate practices relating to BPACR.

## ACKNOWLEDGEMENTS

The original study on which this paper is based was funded by the United States Agency for International Development (USAID), India, vide Grant No. GSM 009 through World Learning, USA.

The opinions, findings, and conclusions or recommendations expressed herein are those of the authors and neither do they necessarily reflect the views of the institutions the authors are affiliated with nor the views of the USAID India.

The authors are grateful to the respondents and NGO partners of Indore and thank other members of the study team: Sandeep Kumar, Dr. S. Kaushik, Dr. Praween Agrawal, Kirti Sangar, Madhvi Mathur, Aashima Garg, Shabnam Verma, Neeraj Verma, and Abhilasha Anand.
